# Most-cited public health articles of scientific journals from Brazil

**DOI:** 10.11606/s1518-8787.2019053001554

**Published:** 2019-09-12

**Authors:** Milena Maria de Araújo Lima Barbosa, Angela Maria Belloni Cuenca, Karoline de Oliveira, Ivan França, Maria do Carmo Avamilano Alvarez, Larissa Yuko Omae

**Affiliations:** I Universidade de São Paulo. Faculdade de Saúde Pública. Programa de Pós-Graduação em Saúde Pública. São Paulo, SP, Brasil; II Universidade de São Paulo. Faculdade de Saúde Pública. Departamento de Saúde, Ciclos de Vida e Sociedade. São Paulo, SP, Brasil; III Universidade de São Paulo. Faculdade de Saúde Pública. Programa Institucional de Bolsas de Iniciação Científica. São Paulo, SP, Brasil; IV Universidade de São Paulo. Faculdade de Saúde Pública. Biblioteca: Centro de Informação e Referência. São Paulo, SP, Brasil; V Universidade de São Paulo. Faculdade de Saúde Pública. Programa Unificado de Bolsas. São Paulo, SP, Brasil

**Keywords:** Periodicals as Topic, Citation Databases, Public Health, Bibliometrics

## Abstract

**OBJECTIVE:**

To describe the most-cited articles in public health scientific journals edited in Brazil.

**METHODS:**

Articles published between 2008 and 2010 by public health journals edited in Brazil and indexed in the Scopus database were included, and citations received up to five years after publication were ranked. We studied a total of 105 articles, as the last seven articles shared the same number of citations and so were given the same rank.

**RESULTS:**

The most-cited articles received a median of 28 citations, and the distribution ranged from 22 to 95 citations. These articles describe advances in the areas of Epidemiology (74%), Health Policies, Planning and Administration (19%), and Social and Human Sciences in Health (7%). Only half mentioned that they have received funding. About 75% of the articles were written by three or more authors and 90%, by authors affiliated to public institutions such as universities and government organizations. Fifteen individuals were responsible for authoring or coauthoring three or more of the 105 articles studied. The journals Cadernos de Saúde Pública, Revista de Saúde Pública, and Ciência & Saúde Coletiva have published the vast majority of the most-cited articles in the area (94%).

**CONCLUSIONS:**

In Brazil, the most-cited articles in public health mainly report Epidemiology research, are written by groups of authors and by researchers affiliated to public institutions and are published in journals with a greater impact. Periodical analyses of these data can show potential changes in the characteristics of articles that most attract public health scientists.

## INTRODUCTION

Citations are considered an indirect measurement of the contribution of an article to the knowledge generated in the field, i.e., the link between the finding of the investigation and its importance for science^1^. Therefore, citations have been widely used and valued to provide indices and impact indicators in science^2^. Garfield and Welljams-Dorof^3^ have identified a significant correlation between the quality and relevance of an article and the number of citations it receives.

By analyzing articles that exert the greatest influence on a field of knowledge, we identified where, how, and by which scientists this field moves forward. Thus, several studies have evaluated highly-cited articles in specific fields such as Rheumatology^4^, Gastroenterology^5^, Psychiatry^6^, Surgery^7^, among others. The most-cited articles represent a high degree of research impact and an effective investment of resources^8^.

Public health is a field of production of multi- and interdisciplinary scientific knowledge^9^, based on epidemiology, social and human sciences in health, and policies, planning and administration in health^10^. This field provides an excellent opportunity to evaluate citation performance among areas. Smith and Leggat^11^ evaluated the 10 most-cited articles in public health, but the authors included articles from only one journal.

In Brazil, few studies have addressed the question of citations, and they are usually from the areas of Information Science, Bibliometrics, and Scientometrics^12^. In specific fields such as public health, some authors, such as Coimbra Jr^15^, Barata^16^, Iriart et al.^10^, Cuenca et al.^17^, have been analyzing the citations attributed to journals and articles, but few of them address the most-cited ones.

This study aimed to characterize the most-cited articles published in public health journals edited in Brazil. Our findings should help students, young researchers, and readers in general to become familiar with the authors, institutions, and journals that have made a leading contribution to public health in Brazil. Also, this study may provide researchers, research funding agencies, and university administrators with insights into the research trends in this field.

## METHODS

Following the trend of bibliometric studies considering the so-called “top 100” of an area of knowledge, we present here the most-cited articles published in Brazilian public health journals.

To assess those articles, we selected the public health scientific journals that are edited in Brazil, according to the *Associação Brasileira de Saúde Coletiva* (*Abrasco* – Brazilian Association of Collective Health), which have been indexed in the Scopus database since 2008. This database was chosen because, besides being one of the main sources of bibliometric data, it indexes more journals edited in Brazil. The initial year was chosen to increase the number of journals represented since fewer journals were edited in Brazil on this database until 2007. Thus, the following journals were selected: Cadernos de Saúde Pública; Ciência & Saúde Coletiva; História, Ciências, Saúde – Manguinhos; Interface: Communicação, Saúde, Educação; Physis; Revista Brasileira de Epidemiologia; Revista de Saúde Pública; and Saúde & Sociedade.

For the retrieval of the articles, we searched for records classified by Scopus as original articles or review articles, published between 2008 and 2010. The final year was considered to include citations received up to five years after publication since citations usually do not occur soon after publication. So, for articles published in 2008, we studied citations received until 2013, and so on. This standardization provides, for all articles, the same time availability to receive citations. Initially, we retrieved 3,242 records and, after the age adjustment, we filtered those articles in descending order of citations and selected the 100 most-cited ones. However, seven papers ranked last in the classification, as they contained the same number of citations (22 citations). Overall, in this study, we included the 105 most cited articles.

We performed the descriptive analysis of the articles included according to the following variables: citation ranking, publication year, publication language, publication type, thematic category, journal title, number of authors (one, two, three, or more), authors’ names, and funding. The type of publication was classified into two stages: first, we evaluated whether the article was methodological or not; second, if it was not methodological, we evaluated whether it was an original study or a review. The thematic addressed in the articles was based on the categories defined by Abrasco for the area of Collective Health, namely: epidemiology; policies, planning, and administration in health; and social and human sciences in health. The first author’s affiliation was classified as a public university, private university, hospital, governmental institution, or non-governmental institution. The funding institutions were categorized into: development agency, such as Research Support Foundations (FAP) and Brazilian National Council for Scientific and Technological Development (CNPq); national governing bodies, such as ministries and state departments; foreign or national institutions, such as World Health Organization; and scientific associations or societies.

## RESULTS

Among the 3,242 articles published between 2008 and 2010 by the journals included in this study, the most-cited articles (n = 105) received between 22 and 95 citations, with a median of 28 (p25 = 24 and p75 = 36). The 105 most-cited articles are listed in the Table 1.

**Table 1:** The most-cited articles of public health scientific journals edited in Brazil.

Rank	Reference	# citations
1	Veras R. Population aging today: Demands, challenges and innovations. Rev Saúde Pública. 2009; 43(3):548-54. http://dx.doi.org/10.1590/S0034-89102009005000025	95
2	Fontanella BJB, Ricas J, Turato ER. Saturation sampling in qualitative health research: Theoretical contributions. Cad Saúde Pública. 2008; 24(1):17-27. http://dx.doi.org/10.1590/S0102-311X2008000100003	85
3	Monteiro CA, Benicio MHD’A, Konno SC, Silva ACF, Lima ALL, Conde WL. Causes for the decline in child under-nutrition in Brazil, 1996-2007. Rev Saúde Pública. 2009; 43(1):35-43. http://dx.doi.org/10.1590/S0034-89102009000100005	76
4	Teixeira MG, Costa MCN, Barreto F, Barreto ML. Dengue: Twenty-five years since reemergence in Brazil. Cad Saúde Pública. 2009; 25(1):S7-S18. http://dx.doi.org/10.1590/S0102-311X2009001300002	67
5	Moura EC, Neto OLM, Malta DC, Moura L, Silva NN, Bernal R, et al. Surveillance of risk-factors for chronic diseases through telephone interviews in 27 Brazilian cities (2006). Rev Bras Epidemiol. 2008; 11(1):20-37. http://dx.doi.org/10.1590/S1415-790X2008000500003	66
6	Ooi E, Gubler DJ. Dengue in Southeast Asia: Epidemiological characteristics and strategic challenges in disease prevention. Cad Saúde Pública. 2008; 25(1):S115-S124. http://dx.doi.org/10.1590/S0102-311X2009001300011	53
7	Coutinho LMS, Scazufca M, Menezes PR. Methods for estimating prevalence ratios in cross-sectional studies. Rev Saúde Pública. 2008; 42(6):992-8. http://dx.doi.org/10.1590/S0034-89102008000600003	49
8	Florindo AA, Hallal PC, Moura EC, Malta DC. Practice of physical activities and associated factors in adults, Brazil, 2006. Rev Saúde Pública. 2009; 43(2):65-73. http://dx.doi.org/10.1590/S0034-89102009000900009	48
9	Monteiro CA, Levy RB, Claro RM, Castro IRR, Cannon G. A new classification of foods based on the extent and purpose of their processing. Cad Saúde Pública. 2010; 26(11):2039-2049. http://dx.doi.org/10.1590/S0102-311X2010001100005	47
10	Maia-Elkhoury ANS, Alves WA, Sousa-Gomes ML, Sena JM, Luna EA. Visceral leishmaniasis in Brazil: Trends and challenges. Cad Saúde Pública. 2008; 24(12):2941-2947. http://dx.doi.org/10.1590/S0102-311X2008001200024	46
11	Passos CJS, Mergler D. Human mercury exposure and adverse health effects in the Amazon: A review. Cad Saúde Pública. 2008; 24(4):S503-S520. http://dx.doi.org/10.1590/S0102-311X2008001600004	46
12	Gigante DP, Moura EC, Sardinha LMV. Prevalence of overweight and obesity and associated factors, Brazil, 2006. Rev Saúde Pública. 2009; 43(2):83-9. http://dx.doi.org/10.1590/S0034-89102009000900011	45
13	Diniz D, Medeiros M. Abortion in Brazil: A household survey using the ballot box technique. Cien Saúde Colet. 2010; 15(1):959-966. http://dx.doi.org/10.1590/S1413-81232010000700002	45
14	Benedetti TRB, Borges LJ, Petroski EL, Gonçalves LHT. Physical activity and mental health status among elderly people. Rev Saúde Pública. 2008; 42(2):302-7. http://dx.doi.org/10.1590/S0034-89102008005000007	44
15	Alves E, Vasconcelos FAG, Calvo MCM, Neves J. Prevalence of symptoms of anorexia nervosa and dissatisfaction with body image among female adolescents in FlorianÃ^3^polis, Santa Catarina State, Brazil . Cad Saúde Pública. 2008; 24(3):503-512. http://dx.doi.org/10.1590/S0102-311X2008000300004	42
16	Giovanella L, Mendonça MHM, Almeida PF, Escorel S, Senna MCM, Fausto MCR, et al. Family health: Limits and possibilities for an integral primary healthcare approach in Brazil. Cien Saúde Colet. 2009; 14(3):783-794. http://dx.doi.org/10.1590/S1413-81232009000300014	42
17	Sarno F, Claro RM, Levy RB, Bandoni DH, Ferreira SRG, Monteiro CA. Estimated sodium intake by the Brazilian population, 2002-2003. Rev Saúde Pública. 2009; 43(2):219-25. http://dx.doi.org/10.1590/S0034-89102009005000002	41
18	Mendes EV. Health care networks. Cien Saúde Colet. 2010; 15(5):2297-2305. http://dx.doi.org/10.1590/S1413-81232010000500005	41
19	Alfradique ME, Bonolo PF, Dourado I, Lima-Costa MF, Macinko J, Mendonça CS, et al. Ambulatory care sensitive hospitalizations: Elaboration of brazilian list as a tool for measuring health system performance (project ICSAP - Brazil). Cad Saúde Publica. 2009; 25(6):1337-1349. http://dx.doi.org/10.1590/S0102-311X2009000600016	40
20	Malta DC, Sardinha LMV, Mendes I, Maria Barreto S, Giatti L, Castro IRR, et al. Prevalence of risk health behavior among adolescents: Results from the 2009 national adolescent school-based health survey (PeNSE). Cien Saúde Colet. 2010; 15(2):3009-3019. http://dx.doi.org/10.1590/S1413-81232010000800002	40
21	Silveira MF, Santos IS, Barros AJD, Matijasevich A, Barros FC, Victora CG. Increase in preterm births in Brazil: Review of population-based studies. Rev Saúde Pública. 2008; 42(5):957-64. http://dx.doi.org/10.1590/S0034-89102008000500023	39
22	Conill EM. A historical and conceptual model for Primary Health Care: Challenges for the organization of primary care and the Family Health Strategy in large Brazilian cities. Cad Saúde Pública. 2008; 24(1):S7-S27. http://dx.doi.org/10.1590/S0102-311X2008001300002	38
23	Duailibi LB, Ribeiro M, Laranjeira R. Profile of cocaine and crack users in Brazil. Cad Saúde Pública. 2008; 24(4):S545-S557. http://dx.doi.org/10.1590/S0102-311X2008001600007	37
24	Ribeiro AQ, Rozenfeld S, Klein CH, Cesar CC, Acurcio FA. Survey on medicine use by elderly retirees in Belo Horizonte, Southeastern Brazil. Rev Saúde Pública. 2008; 42(4):724-32. http://dx.doi.org/10.1590/S0034-89102008005000031	37
25	Chieffi AL, Barata RB. “Judicialization” of public health policy for distribution of medicines. Cad Saúde Pública. 2009; 25(8):1839-1849. http://dx.doi.org/10.1590/S0102-311X2009000800020	37
26	Fernandes LCL, Bertoldi AD, Barros AJD. Health service use in a population covered by the EstratÃ©gia de SaÃºde da FamÃlia (Family Health Strategy). Rev Saúde Pública. 2009; 43(4):595-603. http://dx.doi.org/10.1590/S0034-89102009005000040	36
27	Couto MT, Pinheiro TF, Valença O, Machin R, Silva GSN, Gomes R, et al. Men in primary healthcare: Discussing (in) visibility based on gender perspectives. Interface - Comunic., Saúde, Educ.. 2010; 14(33):257-70. http://dx.doi.org/10.1590/S1414-32832010000200003	36
28	Siqueira FV, Facchini LA, Piccini RX, Tomasi E, Thumé E, Silveira DS, et al. Physical activity in young adults and the elderly in areas covered by primary health care units in municipalities in the South and Northeast of Brazil. Cad Saúde Pública. 2008; 24(1):39-54. http://dx.doi.org/10.1590/S0102-311X2008000100005	35
29	Levy RB, Castro IRR, Cardoso LO, Tavares LF, Sardinha LMV, Gomes FS, et al. Food consumption and eating behavior among brazilian adolescents: National adolescent school-based health survey (PeNSE), 2009. Cien Saúde Colet. 2010; 15(2):3085-3097. http://dx.doi.org/10.1590/S1413-81232010000800013	35
30	Carvalho JAM, Rodríguez-Wong LL. The changing age distribution of the Brazilian population in the first half of the 21st century. Cad Saúde Pública. 2008; 24(3):597-605. http://dx.doi.org/10.1590/S0102-311X2008000300013	34
31	Salvador EP, Florindo AA, Reis RS, Costa EF. Perception of the environment and leisure-time physical activity in the elderly. Rev Saúde Pública. 2009; 43(6):972-80. http://dx.doi.org/10.1590/S0034-89102009005000082	34
32	Seabra AF, Mendonça DM, Thomis MA, Anjos LA, Maia JA. Biological and socio-cultural determinants of physical activity in adolescents. Cad Saúde Pública. 2008; 24(4):721-736. http://dx.doi.org/10.1590/S0102-311X2008000400002	33
33	Barros FC, Victora CG, Scherpbier R, Gwatkin D. Socioeconomic inequities in the health and nutrition of children in low/middle income countries. Rev Saúde Pública. 2010; 44(1):1-16. http://dx.doi.org/10.1590/S0034-89102010000100001	33
34	Souza ECF, Vilar RLA, Rocha NSPD, Uchoa AC, Rocha PM. Primary health care access and receptivity to users: An analysis of perceptions by users and health professionals. Cad Saúde Pública. 2008; 24(1):S100-S110. http://dx.doi.org/10.1590/S0102-311X2008001300015	32
35	Enes CC, Slater B. Obesity in adolescence and its main determinants. Rev Bras Epidemiol. 2010; 13(1):163-71. http://dx.doi.org/10.1590/S1415-790X2010000100015	32
36	Malta M, Cardoso LO, Bastos FI, Magnanini MMF, Silva CMFP. STROBE initiative: guidelines on reporting observational studies. Rev Saúde Pública. 2010; 44(3):559-65. http://dx.doi.org/10.1590/S0034-89102010000300021	32
37	Carret MLV, Fassa ACG, Domingues MR. Inappropriate use of emergency services: A systematic review of prevalence and associated factors. Cad Saúde Pública. 2009; 25(1):7-28. http://dx.doi.org/10.1590/S0102-311X2009000100002	31
38	Coutinho JG, Gentil PC, Toral N. Malnutrition and obesity in Brazil: Dealing with the problem through a unified nutritional agenda. Cad Saúde Pública. 2008; 24(2):S332-S340. http://dx.doi.org/10.1590/S0102-311X2008001400018	31
39	Schlussel MM, Souza EB, Reichenheim ME, Kac G. Physical activity during pregnancy and maternal-child health outcomes: A systematic literature review. Cad Saúde Pública. 2008; 24(4):S531-S544. http://dx.doi.org/10.1590/S0102-311X2008001600006	31
40	Sousa MF, Hamann EM. Family health program in brazil: An incomplete agenda?. Cien Saúde Colet. 2009; 14(1):1325-1335. http://dx.doi.org/10.1590/S1413-81232009000800002	31
41	Lagrotta MTF, Silva WC, Souza-Santos R. Identification of key areas for Aedes aegypti control through geoprocessing in Nova IguaÃ§u, Rio de Janeiro State, Brazil. Cad Saúde Pública. 2008; 24(1):70-80. http://dx.doi.org/10.1590/S0102-311X2008000100007	31
42	Moura FBP, Marques JGW. Folk medicine using animals in the Chapada Diamantina: Incidental medicine?. Cien Saúde Colet. 2008; 13(2):2179-2188. http://dx.doi.org/10.1590/S1413-81232008000900023	30
43	Tesch FC, Oliveira BH, Leão A. Semantic equivalence of the Brazilian version of the Early Childhood Oral Health Impact Scale. Cad Saúde Pública. 2008; 24(8):1897-1909. http://dx.doi.org/10.1590/S0102-311X2008000800018	29
44	Szwarcwald CL, Damacena GN. Complex Sampling Design in Population Surveys: Planning and effects on statistical data analysis. Rev Bras Epidemiol. 2008; 11(1):38-45. http://dx.doi.org/10.1590/S1415-790X2008000500004	29
45	Jaime PC, Figueiredo ICR, Moura EC, Malta DC. Factors associated with fruit and vegetable consumption in Brazil, 2006. Rev Saúde Pública. 2009; 43(2):57-64. http://dx.doi.org/10.1590/S0034-89102009000900008	29
46	Vieira FS. Ministry of health’s spending on drugs: Program trends from 2002 to 2007. Rev Saúde Pública. 2009; 43(4):674-81. http://dx.doi.org/10.1590/S0034-89102009005000041	29
47	Silva Alexandre T, Cordeiro RC, Ramos LR. Factors associated to quality of life in active elderly. Rev Saúde Pública. 2009; 43(4):613-21. http://dx.doi.org/10.1590/S0034-89102009005000030	29
48	Bernal R, Silva NN. Home landline telephone coverage and potential bias in epidemiological surveys. Rev Saúde Pública. 2009; 43(3):421-6. http://dx.doi.org/10.1590/S0034-89102009005000024	29
49	Gonçalves DM, Stein AT, Kapczinski F. Performance of the Self-Reporting Questionnaire as a psychiatric screening questionnaire: A comparativestudy with Structured Clinical Interview for DSM-IV-TR. Cad Saúde Pública. 2008; 24(2):380-390. http://dx.doi.org/10.1590/S0102-311X2008000200017	28
50	Paiva V, Calazans G, Venturi G, Dias R. Age and condom use at first sexual intercourse of Brazilian adolescents. Rev Saúde Pública. 2008; 42(1):45-53. http://dx.doi.org/10.1590/S0034-89102008000800007	28
51	Pinter A, Horta MC, Pacheco RC, Moraes-Filho J, Labruna MB. Serosurvey of Rickettsia spp. in dogs and humans from an endemic area for Brazilian spotted fever in the State of SÃ£o Paulo, Brazil. Cad Saúde Pública. 2008; 24(2):247-252. http://dx.doi.org/10.1590/S0102-311X2008000200003	28
52	Barros FC, Victora CG, Matijasevich A, Santos IS, Horta BL, Silveira MF, et al. Preterm births, low birth weight, and intrauterine growth restriction in three birth cohorts in Southern Brazil: 1982, 1993 and 2004. Cad Saúde Pública. 2008; 24(3):S390-S398. http://dx.doi.org/10.1590/S0102-311X2008001500004	28
53	Silveira VMF, Horta BL. Birth weight and metabolic syndrome in adults: Meta-analysis. Rev Saúde Pública. 2008; 42(1):10-8. http://dx.doi.org/10.1590/S0034-89102008000100002	28
54	Martins AMEBM, Barreto SM, Pordeus IA. Objective and subjective factors related to self-rated oral health among the elderly. Cad Saúde Pública. 2009; 25(2):421-435. http://dx.doi.org/10.1590/S0102-311X2009000200021	28
55	Camargo MBJ, Dumith SC, Barros AJD. Regular use of dental care services by adults: Patterns of utilization and types of services. Cad Saúde Pública. 2009; 25(9):1894-1906. http://dx.doi.org/10.1590/S0102-311X2009000900004	28
56	Menezes G, Aquino EML. Research on abortion in Brazil: Gaps and challenges for the public health field. Cad Saúde Pública. 2009; 25(2):S193-S204. http://dx.doi.org/10.1590/S0102-311X2009001400002	28
57	Schraiber LB, Figueiredo WS, Gomes R, Couto MT, Pinheiro TF, Machin R, et al. Health needs and masculinities: Primary health care services for men. Cad Saúde Pública. 2010; 26(5):961-970. http://dx.doi.org/10.1590/S0102-311X2010000500018	28
58	Araújo CL, Menezes AMB, Vieira MFA, Neutzling MB, Gonçalves H, Anselmi L, et al. The 11-year follow-up of the 1993 Pelotas (Brazil) birth cohort study: Methods. Cad Saúde Pública. 2010; 26(10):1875-1886. http://dx.doi.org/10.1590/S0102-311X2010001000003	28
59	Lino VTS, Pereira SRM, Camacho LAB, Ribeiro Filho ST, Buksman S. Cross-cultural adaptation of the Independence in Activities of Daily Living Index (Katz Index). Cad Saúde Pública. 2008; 24(1):103-112. http://dx.doi.org/10.1590/S0102-311X2008000100010	27
60	Castro IRR, Cardoso LO, Engstrom EM, Levy RB, Monteiro CA. Surveillance of risk factors for non-communicable diseases among adolescents: The experience in Rio de Janeiro, Brazil. Cad Saúde Pública. 2008; 24(10):2279-2288. http://dx.doi.org/10.1590/S0102-311X2008001000009	27
61	Costa CHN. Characterization and speculations on the urbanization of visceral leishmaniasis in Brazil. Cad Saúde Pública. 2008; 24(12):2959-2963. http://dx.doi.org/10.1590/S0102-311X2008001200027	27
62	Figueiredo MD, Campos RO. Mental health in the primary care system of Campinas, SP: Network or spider’s web?. Cien Saúde Colet. 2009; 14(1):129-138. http://dx.doi.org/10.1590/S1413-81232009000100018	26
63	Batista Filho M, Souza AI, Miglioli TC, Santos MC. Anemia and obesity: A paradox of the nutritional transition in Brazil. Cad Saúde Pública. 2008; 24(2):S247-S257. http://dx.doi.org/10.1590/S0102-311X2008001400010	26
64	Barros FC, Victora CG, Horta BL, Gigante DP. Methodology of the Pelotas birth cohort study from 1982 to 2004-5, Southern Brazil. Rev Saúde Pública. 2008; 42(2):7-15. http://dx.doi.org/10.1590/S0034-89102008000900003	26
65	Strauch ES, Pinheiro RT, Silva RA, Horta BL. Alcohol use among adolescents: A population-based study. Rev Saúde Pública. 2009; 43(4):647-55. http://dx.doi.org/10.1590/S0034-89102009005000044	26
66	Oliveira LG, Nappo SA. Characterization of the crack cocaine culture in the city of SÃ£o Paulo: A controlled pattern of use. Rev Saúde Pública. 2008; 42(4):664-71. http://dx.doi.org/10.1590/S0034-89102008005000039	25
67	Oliveira LPM, Assis AMO, Silva MCM, Santana MLP, Santos NS, Pinheiro SMC, et al. Factors associated with overweight and abdominal fat in adults in Salvador, Bahia State, Brazil. Cad Saúde Pública. 2009; 25(3):570-582. http://dx.doi.org/10.1590/S0102-311X2009000300012	25
68	Albuquerque KM, Frias PG, Andrade CLT, Aquino EML, Menezes G, Szwarcwald CL. Pap smear coverage and factors associated with non-participation in cervical cancer screening: An analysis of the Cervical Cancer Prevention Program in Pernambuco State, Brazil. Cad Saúde Pública. 2009; 25(2):S301-309. http://dx.doi.org/10.1590/S0102-311X2009001400012	25
69	Malta DC, Silva MAI, Mello FCM, Monteiro RA, Sardinha LMV, Crespo C, et al. Bullying in Brazilian schools: Results from the national school-based health survey (PeNSE), 2009. Cien Saúde Colet. 2010; 15(2):3065-3076. http://dx.doi.org/10.1590/S1413-81232010000800011	25
70	Tenório MCM, Barros MVG, Tassitano RM, Bezerra J, Tenório JM, Hallal PC. Physical activity and sedentary behavior among adolescent high school students. Rev Bras Epidemiol. 2010; 13(1):105-17. http://dx.doi.org/10.1590/S1415-790X2010000100010	25
71	Schraiber LB, Latorre MRDO, França Jr I, Segri NJ, Lucas D’Oliveira AFP. Validity of the WHO VAW study instrument for estimating gender-based violence against women. Rev Saúde Pública. 2010; 44(4):658-66. http://dx.doi.org/10.1590/S0034-89102010000400009	25
72	Rangel EF, Vilela ML. Lutzomyia longipalpis (Diptera, Psychodidae, Phlebotominae) and urbanization of visceral leishmaniasis in Brazil. Cad Saúde Pública. 2008; 24(12):2948-2952. http://dx.doi.org/10.1590/S0102-311X2008001200025	24
73	Santos AMR, Moura MEB, Nunes BMVT, Leal CFS, Teles JBM. Profile of motorcycle accident victims treated at a public hospital emergency department. Cad Saúde Pública. 2008; 24(8):1927-1938. http://dx.doi.org/10.1590/S0102-311X2008000800021	24
74	Louvison MCP, Lebrão ML, Duarte YAO, Santos JLF, Malik AM, Almeida ES. Inequalities in access to health care services and utilization for the elderly in Sao Paulo, Brazil. Rev Saúde Pública. 2008; 42(4):733-40. http://dx.doi.org/10.1590/S0034-89102008000400021	24
75	Lima CRA, Schramm JMA, Coeli CM, Silva MEM. Review of data quality dimensions and applied methods in the evaluation of health information systems. Cad Saúde Pública. 2009; 25(10):2095-2109. http://dx.doi.org/10.1590/S0102-311X2009001000002	24
76	Munayco CV, Grijalva CG, Culqui DR, Bolarte JL, Suarez-Ognio LA, Quispe N, et al. Outbreak of persistent cutaneous abscesses due to Mycobacterium chelonae after mesotherapy sessions, Lima, Peru. Rev Saúde Pública. 2008; 42(1):146-9. http://dx.doi.org/10.1590/S0034-89102008000100020	24
77	Schmidt MI, Duncan BB, Hoffmann JF, Moura L, Malta DC, Carvalho RMSV. Prevalence of diabetes and hypertension based on selfreported morbidity survey, Brazil, 2006. Rev Saúde Pública. 2009; 43(2):74-82. http://dx.doi.org/10.1590/S0034-89102009000900010	24
78	Moraes ACF, Fulaz CS, Netto-Oliveira ER, Reichert FF. Prevalence of metabolic syndrome in adolescents: A systematic review. Cad Saúde Pública. 2009; 25(6):1195-1202. http://dx.doi.org/10.1590/S0102-311X2009000600002	24
79	Gonçalves CV, Cesar JA, Mendoza-Sassi RA. Quality and equity in prenatal care: A population-based study in southern Brazil. Cad Saúde Pública. 2009; 25(11):2507-2516. http://dx.doi.org/10.1590/S0102-311X2009001100020	24
80	Araújo CS, Lima RC, Peres MA, Barros AJD. Use of dental services and associated factors: A population-based study in southern Brazil. Cad Saúde Pública. 2009; 25(5):1063-1072. http://dx.doi.org/10.1590/S0102-311X2009000500013	24
81	Castro HA, Cunha MF, Mendonça GAS, Junger WL, Cunha-Cruz J, Leon AP. Effect of air pollution on lung function in schoolchildren in Rio de Janeiro, Brazil. Rev Saúde Pública. 2009; 43(1):26-34. http://dx.doi.org/10.1590/S0034-89102009000100004	24
82	Lima LHM, Viana MC. Prevalence and risk factors for HIV, syphilis, hepatitis B, hepatitis C, and HTLV-I/II infection in low-income postpartum and pregnant women in Greater Metropolitan VitÃ^3^ria, EspÃrito Santo State, Brazil. Cad Saúde Pública. 2009; 25(3):668-676. http://dx.doi.org/10.1590/S0102-311X2009000300021	24
83	Ignotti E, Valente JG, Longo KM, Freitas SR, Hacon SS, Netto PA. Impact on human health of particulate matter emitted from burnings in the Brazilian Amazon region. Rev Saúde Pública. 2010; 44(1):121-30. http://dx.doi.org/10.1590/S0034-89102010000100013	24
84	Mitre SM, Siqueira-Batista R, Girardi-de-Mendonça JM, Morais-Pinto NM, Meirelles CAB, Pinto-Porto C, et al. Active teaching-learning methodologies in health education: Current debates. Cien Saúde Colet. 2008; 13(2):2133-2144. http://dx.doi.org/10.1590/S1413-81232008000900018	23
85	Giacomin KC, Peixoto SV, Uchoa E, Lima-Costa MF. A population-based study on factors associated with functional disability among older adults in the Great Metropolitan Belo Horizonte, Minas Gerais State, Brazil. Cad Saúde Pública. 2008; 24(6):1260-1270. http://dx.doi.org/10.1590/S0102-311X2008000600007	23
86	Paniz VMV, Fassa AG, Facchini LA, Bertoldi AD, Piccini RX, Tomasi E, et al. Access to continuous-use medication among adults and the elderly in South and Northeast Brazil. Cad Saúde Pública. 2008; 24(2):267-280. http://dx.doi.org/10.1590/S0102-311X2008000200005	23
87	Ronzani TM, Silva CM. Brazil’s Family Health program according to healthcare practitioners, managers and users. Cien Saúde Colet. 2008; 13(1):23-34. http://dx.doi.org/10.1590/S1413-81232008000100007	23
88	Tanaka OY, Ribeiro EL. Mental health in primary care: Ways to reach an integral care. Cien Saúde Colet. 2009; 14(2):477-486. http://dx.doi.org/10.1590/S1413-81232009000200016	23
89	Caprara A, Lima JWO, Marinho ACP, Calvasina PG, Landim LP, Sommerfeld J. Irregular water supply, household usage and dengue: A bio-social study in the Brazilian Northeast. Cad Saúde Pública. 2009; 25(1):S125-S136. http://dx.doi.org/10.1590/S0102-311X2009001300012	23
90	Lopes CLR, Teles SA, Espírito-Santo MP, Lampe E, Rodrigues FP, Motta-Castro ARC, et al. Prevalence, risk factors and genotypes of hepatitis C virus infection among drug users, Central-Western Brazil. Rev Saúde Pública. 2009; 43(1):43-50. http://dx.doi.org/10.1590/S0034-89102009000800008	23
91	Scatena LM, Villa TCS, Netto AR, Kritski AL, Figueiredo TMRM, Vendramini SHF, et al. Difficulties in the accessibility to health services for tuberculosis diagnoses in Brazilian municipalities. Rev Saúde Pública. 2009; 43(3):389-97. http://dx.doi.org/10.1590/S0034-89102009005000022	23
92	Chrestani MAD, Santos IS, Matijasevich AM. Self-reported hypertension: Validation in a representative cross-sectional survey. Cad Saúde Pública. 2009; 25(11):2395-2406. http://dx.doi.org/10.1590/S0102-311X2009001100010	23
93	Borges CQ, Silva RCR, Assis AMO, Pinto EJ, Fiaccone RL, Pinheiro SMC. Factors associated with anemia in children and adolescents in public schools in Salvador, Bahia State, Brazil. Cad Saúde Pública. 2009; 25(4):877-888. http://dx.doi.org/10.1590/S0102-311X2009000400019	23
94	Santos MS, Hino AAF, Reis RS, Rodriguez-Anez CR. Prevalence of barriers for physical activity in adolescents. Rev Bras Epidemiol. 2010; 13(1):94-104. http://dx.doi.org/10.1590/S1415-790X2010000100009	23
95	Lima ALL, Silva ACF, Konno SC, Conde WL, Benicio MHDA, Monteiro CA. Causes of the accelerated decline in child undernutrition in Northeastern Brazil (1986-1996-2006). Rev Saúde Pública. 2010; 44(1):17-27. http://dx.doi.org/10.1590/S0034-89102010000100002	23
96	Ayres ARG, Silva GA. Cervical HPV infection in Brazil: Systematic review. Rev Saúde Pública. 2010; 44(5):963-74. http://dx.doi.org/10.1590/S0034-89102010000500023	23
97	Antunes JLF, Narvai PC. Dental health policies in Brazil and their impact on health inequalities. Rev Saúde Pública. 2010; 44(2):360-5. http://dx.doi.org/10.1590/S0034-89102010005000002	23
98	Chor D, Werneck GL, Faerstein E, Alves MGM, Rotenberg L. The Brazilian version of the effort-reward imbalance questionnaire to assess job stress. Cad Saúde Pública. 2008; 24(1):219-224. http://dx.doi.org/10.1590/S0102-311X2008000100022	22
99	Monteiro CA, Florindo AA, Claro RM, Moura EC. Validity of indicators of physical activity and sedentariness obtained by telephone survey. Rev Saúde Pública. 2008; 42(4):575-81. http://dx.doi.org/10.1590/S0034-89102008000400001	22
100	Barreto SM, Figueiredo RC. Chronic diseases, self-perceived health status and health risk behaviors: Gender differences. Rev Saúde Pública. 2009; 43(2):38-47. http://dx.doi.org/10.1590/S0034-89102009000900006	22
101	Pepe VLE, Ventura M, Sant’ana JMB, Figueiredo TA, Souza VR, Simas L, et al. Characterization of lawsuits for the supply of “essential” medicines in the State of Rio de Janeiro, Brazil. Cad Saúde Pública. 2010; 26(3):461-471. http://dx.doi.org/10.1590/S0102-311X2010000300004	22
102	Bezerra IN, Sichieri R. Characteristics and spending on out-of-home eating in Brazil. Rev Saúde Pública. 2010; 44(2):221-9. http://dx.doi.org/10.1590/S0034-89102010000200001	22
103	Galduroz JCF, Sanchez ZVDM, Opaleye ES, Noto AR, Fonseca AM, Gomes PLS, et al. Factors associated with heavy alcohol use among students in Brazilian capitals. Rev Saúde Pública. 2010; 44(2):267-73. http://dx.doi.org/10.1590/S0034-89102010000200006	22
104	Clemens SAC, Farhat CK. Seroprevalence of herpes simplex 1-2 antibodies in Brazil. Rev Saúde Pública. 2010; 44(4):726-34. http://dx.doi.org/10.1590/S0034-89102010000400017	22
105	Silva AAM, Silva LM, Barbieri MA, Bettiol H, Carvalho LM, Ribeiro VS, et al. The epidemiologic paradox of low birth weight in Brazil. Rev Saúde Pública. 2010; 44(5):767-75. http://dx.doi.org/10.1590/S0034-89102010005000033	22

As described in Table 2, of the 105 articles, most were original articles (70%), in the area of Epidemiology (74%), written by three or more authors (74%) and published in English (57%). All have abstracts in Portuguese and English, and 34 (32%) also have an Abstract in Spanish. It is worth noting that, despite standardizing the citation window (citations up to five years after publication), we identified a few of the most-cited articles published in 2010.

**Table 2:** Characteristics of the most-cited articles in the public health area between 2008 and 2010. (n = 105)

Characteristics	n	%
Publication year		
2008	42	40
2009	39	37
2010	24	23
# authors		
1	5	5
2	22	21
3 or more	78	74
Language		
Portuguese	45	43
English and Portuguese	36	34
English	24	23
Area		
Epidemiology	78	74
Policies, Planning, and Management	20	19
Social and Human Sciencies in Health	7	7
Article type		
Original	73	70
Methodological	17	16
Review	15	14
Funding		
Yes	52	50
No	53	50

Cadernos de Saúde Pública, Revista de Saúde Pública, and Ciência & Saúde Coletiva have published the vast majority of the most-cited articles in the area (94%). Another group is constituted by the Revista Brasileira de Epidemiologia, Interface, História, Ciência & Saúde, Physis, and Saúde e Sociedade, the articles in which seldom if ever feature among the most-cited ones. The distribution of the most-cited articles according to the journal is shown in Table 3.

**Table 3:** Distribution of the most-cited articles by public health journal. (n = 105)

Journal (Institution)	Most-cited articles	

	n	%
Cad Saúde Pública (Fiocruz)	48	46
Rev Saúde Pública (USP/FSP)	39	37
Ciênc & Saúde Coletiva (Abrasco)	12	11
Rev Bras Epidemiol (Abrasco)	5	5
Interface (Unesp)	1	1
Hist Ciênc Saúde Manguinhos (Fiocruz)	0	0
Physis (UERJ)	0	0
Saúde e Sociedade (USP/FSP and APSP)	0	0

**Total**	**105**	**100**

Regarding the affiliations of the first author of each article (Table 4), public universities represented 67% of the institutions. Authors were also affiliated to governmental organizations (23%) – such as the Fiocruz Foundation, the Ministry of Health and health departments – and to private undergraduate institutions (6%).

**Table 4:** Institutional affiliation of the first author of ≥ 3 the most-cited articles published by public health journals edited in Brazil between 2008 and 2010. (n = 105)

Institution	Category	n	%
Universidade de São Paulo	Public university	23	22
Fiocruz-Fundação Oswaldo Cruz	Governmental institution	11	11
Universidade Federal de Pelotas	Public university	10	10
Ministério da Saúde (Brazil)	Governmental institution	6	6
Universidade Estadual do Rio de Janeiro	Public university	4	4
Universidade Federal da Bahia	Public university	4	4
Universidade Federal de Minas Gerais	Public university	4	4
Universidade Católica de Pelotas	Private university	4	4
Universidade Federal de Santa Catarina	Public university	3	3
Universidade Federal de São Paulo	Public university	3	3
Universidade Federal do Rio Grande do Sul	Public university	3	3

Regarding research funding, 52 articles reported some kind of funding; of these, half reported support from two or more institutions. The most frequently mentioned funding agencies or institutions were FAP (35/52) and CNPq (31/52). Support from foreign agencies was mentioned in 25 of the 52 articles (48%). Brazilian government agencies accounted for 21% (11/52) of these grants, and a research incentive association accounted for 1 grant (data not shown in table).

In relation to authorship and coauthorship, in total, 388 individuals were responsible for authoring or coauthoring these articles. Table 5 shows the most-productive researchers (n = 15). In addition, eight other researchers were responsible for 1 authorship and 1 coauthorship; 26, for two coauthorships; 84, for 1 authorship; and 255, for 1 coauthorship (data not shown in table).

**Table 5:** Authors who contributed to ≥ 3 most-cited articles of public health journals edited in Brazil and published between 2008 and 2010, according to authorship, coauthorship and the sum of both.

Author	ResearcherID	Authoring	Coauthoring	Total
Monteiro, Carlos Augusto	F-9892-2012	3	4	7
Malta, Deborah Carvalho	H-7880-2012	2	4	6
Barros, Aluísio Jardim Dornellas	A-7417-2008	0	5	5
Barros, Fernando C.	D-4857-2013	3	1	4
Castro, Inês Rugani Ribeiro	-	1	3	4
Levy, Renata Bertazzi	F-8931-2012	1	3	4
Claro, Rafael Moreira	F-8996-2012	0	4	4
Hallal, Pedro Curi	A-3249-2011	0	4	4
Horta, Bernardo Lessa	A-7604-2008	0	4	4
Sardinha, Luciana Monteiro Vasconcelos	-	0	4	4
Victora, Cesar Gomes	D-4476-2013	0	4	4
Schraiber, Lilia Blima	B-5708-2014	2	1	3
Florindo, Alex Antonio	K-1870-2013	1	2	3
Moura, Erly Catarina	-	0	3	3
Moura, Lenildo	-	0	3	3

## DISCUSSION

The number of citations received by public health articles differs significantly from that found by studies in diverse subjects and countries, varying from about four thousand citations received^5^ to one thousand^18^, from the medical field, but over an extended period, stretching back to the 1950s. In a bibliometric study of systematic reviews, Royle et al.^19^ found an average of 26.5 citations after four years of publication. The authors used the same information source, the Scopus database, and a similar analysis period (year of 2008) to that of our study. In our study, median citations were equal to 28. Although we included a period longer than that of Royle et al.^19^, which could overestimate the total citations of the articles of our study compared with the one of Royle et al.^19^, this overestimation would be counterbalanced since systematic reviews tend to receive more citations^20,21^. In fact, the number of citations potentially reflects the process of creation and dissemination, which differs between the areas of knowledge^22^, the periods covered and the data sources, among other factors.

Less than 5% of the most-cited articles have only one author, while most (74%) have three or more authors. This result corroborates the literature on the positive influence of the number of authors per article on the number of citations received^23^. Regarding language, more than half were published in English (part of them, bilingual). Clearly, it denotes the effort for internationalization by the journals edited in Brazil.

The proportion of the most-cited articles reduced from 2008 to 2010. Certainly, the more recent, the less time the article would be found, read and quoted more often and by more authors. However, for all articles, we counted citations received up to five years after publication, reducing the bias of publication year. Thus, this result may have been due to the number of article journals published during the years considered in this study. In fact, the two journals that contributed the most to the list of the most-cited articles published a total of 605 articles in 2008, 521 in 2009, and 422 in 2010; that is, the number of most-cited articles decreased as the total of articles also decreased.

As for the influence of the impact factor of journals on the citations, there are controversies. According to Callaham et al.^24^, the greater the impact of the journal, the more its articles are cited. The authors reported the impact factor is more influential even than variables related to the quality of the investigation, such as the study design and other methodological aspects. As a matter of fact, the two journals with the highest impact factors (SJR 2010 for Cadernos de Saúde Pública = 0.788 and for Revista de Saúde Pública = 0.817) published 83% of the most-cited articles between 2008 and 2010. Shadgan et al.^18^, when evaluating the most-cited articles in the medical field, also found most of these were published by journals with a high impact factor. For Larivière and Gingras^25^, this influence can result both from the better dissemination of the journals that have a greater impact, and from the citing researchers’ perception that the articles published in these journals have higher quality.

Slyder et al.^2^, however, emphasize that being published by high impact journals does not guarantee that the article is highly cited since the citation rate varies greatly between articles. This indicates that other features in these articles make them attract more citations. The study by Royle et al.^19^ corroborates this information by showing that, although impact metrics have explained more than half of the variation in citations, they do not accurately represent the number of citations of individual articles.

It should be noted that, when analyzing the journals in which the most-cited articles were published, a consistency can be seen between the order of the most-cited articles and the number of those that did not receive any citations^17^, i.e., the journals that published more articles with the highest number of citations were also the ones that published fewer articles that have never been cited.

Regarding the sub-areas, Iriart et al.^10^, in a recent evaluation of the scientific production of postgraduate programs in the public health area, found a similar tendency in the proportion of researchers in each discipline: 49% belong to Epidemiology; 20%, to Health Policies, Planning and Management; and 17%, to the Social and Human Sciences in Health. This proportion is reflected, in this study, by the distribution of the most-cited articles according to the sub-areas.

The search for institutional excellence has aroused interest in identifying the institutions performing best in the development of science in a country, or in a specific area. In public health in Brazil, public universities stand out as science generators. Packer and Meneghini^12^, in 2006, had already identified the influence of public universities on the impact of articles. Both in this paper and in the study by Packer and Meneghini^12^, the presence of private higher education institutions in the most-cited articles was also observed, although to a lesser extent.

In Brazil, public health research is subsidized by development agencies, national government agencies and mainly by public resources^26,27^, as corroborated by our study. We can infer that this is a characteristic of developing countries, such as Brazil, and of a multidisciplinary area, such as public health, while private financing is correlated with the highest citation index in the area of cardiology in developed countries^28^. However, some journals do not adopt the requirement of funding acknowledgments, and this fact may have led to the large proportion of articles classified as being without financial support^27^.

As for the type of article among the most-cited, we selected a priori the categories original articles and reviews to ensure research articles, supposing that editorials, opinions, comments, among others are not expected to receive enough citations to be among the most-cited publications. In this study, we found an opinion article among the most-cited, besides debates, forums, and notes, which were then categorized by the authors according to the classifications considered in this investigation (original, methodological, or review). This is an error that can be attributed to the indexer or editor of the journal. This is a limitation of our study, since other records not indexed as original articles or reviews may have had more citations than the 105 articles included here.

Another limitation is that we used only the Scopus database in data collection. However, this is the database that indexes most journals of public health edited in Brazil, which is why it was chosen. Also, our findings are limited by the fact that we have not assessed the quality of the citations.

Additionally, we emphasize that the object of this study was the scientific journals edited in Brazil and their respective articles. As the literature on public health tends to be dispersed in journals from other areas (Medicine, Psychology, Oral Health, etc.), our findings are limited to a part of the Brazilian scientific production. Future investigation evaluating public health articles published by journals from other areas of knowledge may provide a broader picture of the scientific production in this area.

## CONCLUSION

This panorama of the most-cited articles in the field of public health helps us to understand the science produced in Brazil in this field of knowledge. The most frequently cited articles are written by groups of authors and researchers affiliated to public institutions, belong to the Epidemiology sub-area, and are published in journals that have a greater impact. This may inspire new generations of scientists as to the characteristics that will potentially increase the impact of their research. Periodical analyses such as these can show possible changes in the characteristics of articles that most attract public health scientists. Future investigation assessing the citing articles of public health journals could clarify the quality of the citations. Also, future studies evaluating public health articles published by journals from other areas of knowledge may provide a broader picture of the scientific production in this area.

## References

[1] 1. Hamilton DP. Publishing by-- and for? -- the Numbers: new evidence raises the possibility that a majority of scientific papers make negligible contributions to knowledge. Science.1990;250(4986):1331-2. 10.1126/Science.2255902 2255902

[2] 2. Slyder JB, Stein BR, Sams BS, Walker DM, Bealey BJ, Feldhaus JJ, et al. Citation pattern and lifespan: a comparison of discipline, institution, and individual. Scientometrics. 2011;89:955. 10.1007/s11192-011-0467-x

[3] 3. Garfield E, Welljams-Dorof A. Of noble class: a citation perspective on high impact research authors. Theor Med. 1992;13(2):117-35. 10.1007/BF02163625 1412072

[4] 4. Perez-Cabezas V, Ruiz-Molinero C, Carmona-Barrientos I, Herrera-Viedma E, Cobo MJ, Moral-Munoz JA. Highly cited papers in rheumatology: identification and conceptual analysis. Scientometrics. 2018;116(1):555-68. 10.1007/s11192-018-2712-z 30247554

[5] 5. Tang X, Gong W, Yuan F, Li R, Han X, Huang S, et al. Top-cited articles in digestive system disease from 1950 to 2013. J Gastroenterol Hepatol. 2016;31(1):107-11. 10.1111/jgh.13032 26173467

[6] 6. Mazhari S. The 100 top-cited articles published in psychiatry journals. J Psychiatr Pract. 2013;19(4):327-38. 10.1097/01.pra.0000432604.06835.da 23852109

[7] 7. Bayley M, Brooks F, Tong A, Hariharan K. The 100 most cited papers in foot and ankle surgery. Foot (Edinb). 2014;24(1):11-6. 10.1016/j.foot.2013.11.003 24316021

[8] 8. Ranasinghe I, Shojaee A, Bikdeli B, Gupta A, Chen R, Ross JS, et al. Poorly cited articles in peer-reviewed cardiovascular journals from 1997 to 2007: analysis of 5-year citation rates. Circulation. 2015;131(20):1755-62. 10.1161/CIRCULATIONAHA.114.015080 PMC4560203 25812573

[9] 9. Alvarenga AT, Sommerman A, Alvarez AMS. Congressos internacionais sobre transdisciplinaridade: reflexões sobre emergências e convergências de ideias e ideais na direção de uma nova ciência moderna. Saude Soc. 2005;14(3):9-29. 10.1590/S0104-12902005000300003

[10] 10. Iriart JAB, Deslandes SF, Martin D, Carmargo Jr KR, Carvalho MS, Coeli CM. A avaliação da produção científica nas subáreas da Saúde Coletiva: limites do atual modelo e contribuições para o debate. Cad Saude Publica. 2015;31(10):2137-47. 10.1590/0102-311X00065515 26735381

[11] 11. Smith DR, Leggat PA. Ten citation classics from the Australian and New Zealand Journal of Public Health. Aust N Z J Public Health. 2008;32(2):105-6. 10.1111/j.1753-6405.2008.00183.x 18412677

[12] 12. Packer AL, Meneghini R. Articles with authors affliliated to Brazilian institutions published from 1994 to 2003 with 100 or more citations: I The weight of international collaboration and the role of the networks. An Acad Bras Cienc. 2006;78(4):841-53. 10.1590/S0001-37652006000400017 17143417

[13] 13. Leta J. Brazilian growth in the mainstream science: the role of human resources and national journals. J Scientometric Res.2012;1(1):44-52. 10.5530/jscires.2012.1.9

[14] 14. Mugnaini R, Digiampetri LA, Mena-Chalco JP. Comunicação científica no Brasil (1998-2012): indexação, crescimento, fluxo e dispersão. Transinformação. 2014;26(3):239-52. 10.1590/0103-3786201400030002

[15] 15. Coimbra Jr CEA. Produção científica e impacto em Saúde Coletiva. Cad Saude Publica. 2004;20(4):878-9. 10.1590/S0102-311X2004000400001

[16] 16. Barata RB. SciELO Saúde Pública: o desempenho dos Cadernos de Saúde Pública e da Revista de Saúde Pública. Cad Saude Publica. 2007;23(12):3031-40. 10.1590/S0102-311X2007001200025 18157346

[17] 17. Cuenca AMB, Barbosa MMAL, Oliveira K, Quinta FP, Alvarez MCA, França Jr I. Artigos não citados nas revistas brasileiras em Saúde Pública. Rev Saude Publica. 2017;51:114. 10.11606/s1518-8787.2017051000442 PMC5708265 29211202

[18] 18. Shadgan B, Roig M, HajGhambari B, Reid WD. Top-cited articles in rehabilitation. Arch Phys Med Rehabil. 2010;91(5):806-15. 10.1016/j.apmr.2010.01.011 20434622

[19] 19. Royle P, Kandala NB, Barnard K, Waugh N. Bibliometrics of systematic reviews: analysis of citation rates and journal impact factors. Systematic Rev. 2013;2:74. 10.1186/2046-4053-2-74 PMC3847500 24028376

[20] 20. Patsopoulos NA, Analatos AA, Ioannidis JPA. Relative citation impact of various study designs in the Health Sciences. JAMA. 2005;293(19):2362-6. 10.1001/jama.293.19.2362 15900006

[21] 21. Bhandari M, Busse J, Devereaux PJ, Montori VM, Swiontkowski M, Tornetta P, III, et al. Factors associated with citation rates in the orthopedic literature. Can J Surg. 2007 [cited 2017 Febr 15];50(2):119-23. Available from: https://www.ncbi.nlm.nih.gov/pmc/articles/PMC2384258/ PMC2384258 17550715

[22] 22. Pendlebury DA. Science, citation and funding [letter to the editor]. Science. 1991 [cited 2017 Febr 15];251:1410-1. Available from: http://garfield.library.upenn.edu/papers/pendleburyscience1991.html 10.1126/science.251.5000.1410-b 17779411

[23] 23. Figg WD, Dunn L, Liewehr DJ, Steinberg SM, Thurman PW, Barrett JC, et al. Scientific collaboration results in higher citations rates of published articles. Pharmacotherapy. 2006;26(6):759-67. 10.1592/phco.26.6.759 16716129

[24] 24. Callaham M, Wears RL, Webwe E. Journal prestige, publication bias, and other characteristics associated with citation of published studies in peer-reviewed journals. JAMA. 2002;287(21):2847-50. 10.1001/jama.287.21.2847 12038930

[25] 25. Larivière V, Gingras Y. The impact factor’s Matthew effect: a natural experiment in Bibliometrics. J Am Soc Inf Sci Technol. 2010;61(2):424-7. 10.1002/asi.21232

[26] 26. Guimarães R. Pesquisa em saúde no Brasil: contexto e desafios. Rev Saude Publica. 2006; 40 nº espec:3-10. 10.1590/S0034-8910200600040000 16924297

[27] 27. Cuenca AMB, Noronha DP, Ueno HM, Kobayashi KM. Periódicos brasileiros de saúde pública: a questão do financiamento. INCID Rev Cienc Inf Doc. 2011;2(2):101-10. 10.11606/issn.2178-2075.v2i2p101-110

[28] 28. Khan MS, Usman MS, Fatima K, Hashmani N, Siddiqi TJ, Riaz H, et al. Characteristics of highly cited articles in interventional cardiology. Am J Cardiol. 2017;120(11):2100-9. 10.1016/j.amjcard.2017.08.030 28964383

